# Preattentive and Predictive Processing of Visual Motion

**DOI:** 10.1038/s41598-018-30832-9

**Published:** 2018-08-17

**Authors:** Constanze Schmitt, Steffen Klingenhoefer, Frank Bremmer

**Affiliations:** 1 Dept. Neurophysics, Philipps-Universität Marburg Karl-von-Frisch Str 8a, Marburg, D-35043 Germany; 2 Center for Mind, Brain and Behavior - CMBB, Hans-Meerwein-Straße 6, Marburg, D-35032 Germany; 3 Center for Molecular and Behavioral Science (CMBN), Rutgers University, Newark, NJ USA

## Abstract

Interaction with the environment requires fast and reliable sensory processing. The visual system is confronted with a continuous flow of high-dimensional input (e.g. orientation, color, motion). From a theoretical point of view, it would be advantageous if critical information was processed independent of attentional load, i.e. preattentively. Here, we hypothesized that visual motion is such a critical signal and aimed for a neural signature of its preattentive encoding. Furthermore, we were interested in the neural correlates of predictability of linear motion trajectories based on the presence or absence of preceding motion. We presented a visual oddball paradigm and studied event-related potentials (ERPs). Stimuli were linearly moving Gabor patches that disappeared behind an occluder. The difference between deviant and standard trials was a trajectory change which happened behind the occluder in deviant trials only, inducing a prediction error. As hypothesized, we found a visual mismatch negativity-component over parietal and occipital electrodes. In a further condition, trials without preceding motion were presented in which the patch just appeared from behind the occluder and, hence, was not predictable. We found larger ERP-components for unpredictable stimuli. In summary, our results provide evidence for a preattentive and predictive processing of linear trajectories of visual motion.

## Introduction

The perception of visual motion is of critical importance for interaction with the environment. As an example, interceptive movements like catching a flying ball suggest that our visual system not only encodes visual motion veridically, but also anticipates or predicts motion^1^. During everyday life, however, the visual system has to process numerous visual features in parallel, which all compete for processing resources. Hence, from a theoretical perspective, it would be beneficial if critical motion information or sudden trajectory changes were detected to a certain degree automatically, i.e. preattentively, by our visual system.

The visual mismatch negativity (vMMN), a specific component of event-related potentials (ERPs), can be used to test for such preattentive processing of sensory stimuli^2^. The MMN is typically shown in oddball experiments and reflects a prediction error by the encoding of unpredicted infrequent deviant stimuli interrupting a sequence of frequent standard stimuli^3,4^. The MMN is defined by the difference in ERPs elicited by standard and deviant stimuli; in particular, the N2-component becomes more negative for deviant as compared to standard trials^5^. The resulting negative component in the difference, the MMN, typically reveals its peak with a posterior scalp distribution in a time window between 150 ms and 400 ms after the sensory event^3^.

For many years, the MMN was described only in the auditory domain^6,7^ and it was discussed controversially whether or not a MMN existed for other sensory modalities, too. In recent years, however, several groups have also observed visual MMNs^8–11^. In these studies, the MMN was shown for many different visual stimulus features like orientation^10,12,13^, direction of motion^14,15^, duration^16^, color^17–19^, numerosity^9^ and also for higher cognitive features such as object structure^20^ and facial emotions^21,22^. While the occurrence of visual MMNs is now rather undisputed, the discussion about its neural basis continues to be controversial. The currently most reasonable idea to describe the generation of visual MMN is the prediction-error account^3^. The frequently presented standard stimuli are used to extract an abstract sequential rule. Based on this information a prediction (a predictive model) about the forthcoming visual event is created which is compared to the actual visual stimulation. In cases of an unexpected deviant stimulus the expectancy (or prediction) is violated which results in the generation of the MMN. This idea is in line with the concept of predictive coding and accordingly is considered a perceptual prediction error signal^4,23^. Furthermore, the MMN is considered a characteristic feature of automatic or preattentive processing of sensory signals^20,24–26^. This view was deduced from the finding that the MMN occurs also in experimental paradigms in which the participants have to perform a task which is unrelated to the stimulus feature. In such a case the participants’ attention is directed away from the MMN stimulus or feature in question^14,21,27,28^.

In addition to investigating the neural correlate of perceptual prediction errors, our study was also concerned with a neural correlate of predictability of motion trajectories *per se*. It is known that the predictability of a moving target’s trajectory is reflected in specific components of the ERP. If a moving target is occluded for a certain time, the ERPs induced by its reappearance reveal differences dependent on whether the reappearance times and locations can be correctly predicted or not^29^. The early P1-component shows larger values if the target appears at the predicted location^30^ and/or the predicted time^29,31^. It is unknown, however, if and how these ERP-components are related to the vMMN.

Hence, in our current study we aimed to compare the neural correlates of both, prediction error and predictability, in the same subjects. In a visual oddball paradigm, we compared EEG responses to frequently shown standard trials with those of deviant trials. The stimulus was a moving Gabor pattern which, in deviant trials, moved along a new trajectory upon reappearance after an occlusion phase. We hypothesized that motion trajectories are processed in an automatic or preattentive manner that is reflected in a MMN-component and can be observed in the reappearance ERPs. In a second step, we compared the ERPs of trials with different levels of predictability. In particular, we again used the reappearance ERPs from standard trials as a condition that is perfectly predictable assuming a steady linear target motion. As a comparison condition that was physically identical, but unpredictable, we measured onset ERPs evoked by the appearance of the moving target at random positions next to the occluder.

## Results

To direct the participants’ focus of attention, they were involved in a task that required the detection of a subtle change of the spatial frequency of either the central fixation target or the peripheral moving target, which happened in 10% of all trials. Mean reaction times for detecting the changes were 516 ms (std: 44 ms) for the central stationary target and 591 ms (std: 51 ms) for the peripheral moving target. The accuracy for discriminating whether the spatial frequency of the Gabor pattern changed to a higher or lower spatial frequency were 77.8% (std: 16.3%) for the central and 97.6% ± 1.5% for the peripheral target. The differences in the reaction times and accuracies for the two attention conditions were most likely caused by the differently sized Gabor patterns. As described in the methods section, the moving peripheral Gabor pattern was larger (twice the size) than the central pattern. By increasing the size of the peripheral pattern, we aimed for similar levels of difficulty in the two attention conditions. Our participants revealed a typical speed-accuracy tradeoff in their behavior with faster but less precise responses to the central target.

### The effect of discontinuities in target trajectories – a Mismatch Negativity (MMN)

In this first part of our analysis we only consider data collected in the behavioral condition “central”. In a later step, we are going to compare data from both behavioral conditions, i.e. central vs. peripheral. Unless otherwise noted, we are considering the ERPs evoked by (re)appearance of the moving target from behind the occluder in the following. This (re)appearance of the moving target elicited a typical sequence of visual ERP-components: C1 peaking around 65 ms, P1 peaking around 95 ms, N2 peaking around 140 ms and P2 peaking around 215 ms after target (re)appearance. The two conditions “above” and “below” yielded comparable results. Therefore, the data from these two conditions were considered together, i.e. averaged for further analyses. Inspection of the “half trajectory, disappear” condition confirmed that no late potentials evoked by the disappearing target interfered with the reappearance ERPs in the “full trajectory” conditions (data not shown).

**Figure 1:**
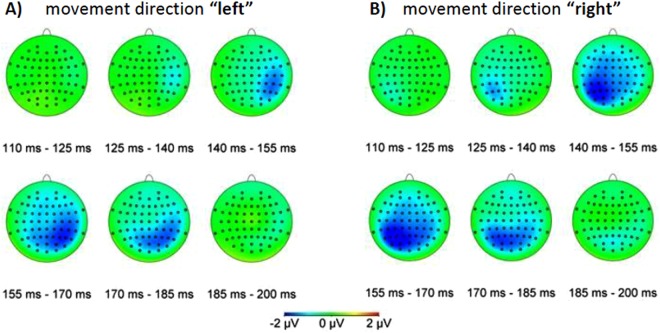
Difference between standard and deviant ERPs for trials in the “central” attention condition. Each plot shows ERP_Deviant_ − ERP_Standard_, averaged over 15 ms, for different times relative to the reappearance of the moving target. (**A**) target reappearance in the left half of the visual field (“left”); (**B**) target re-appearance in the right half of the visual field (“right”). In both cases the “above” and “below” conditions were averaged. Topographic maps show the differential EEG response signals, with blue indicating negative values.

In Fig. 1 the difference between the standard and deviant condition (standard data subtracted from deviant data, or ERP_Deviant_ − ERP_Standard_) is presented in the time window from 110 ms to 200 ms in slices of 15 ms. The different topographic maps show the temporal evolution of the MMN signal with a peak at around 160 ms. This value is perfectly in line with data from the literature^3^. Data were collected in the “central” condition, i.e. when participants were performing the spatial frequency change detection task for the central fixation target. Note that the visual stimulation upon reappearance was identical for deviant and standard trials and they only differed in their earlier trajectories. Figure 1 clearly shows for both movement directions, left (A) and right (B), respectively, a negative component that peaks around 160 ms over parieto-occipital electrodes contralateral to the position of the target’s reappearance. In the following we will refer to this component as a Mismatch Negativity (MMN).

**Figure 2:**
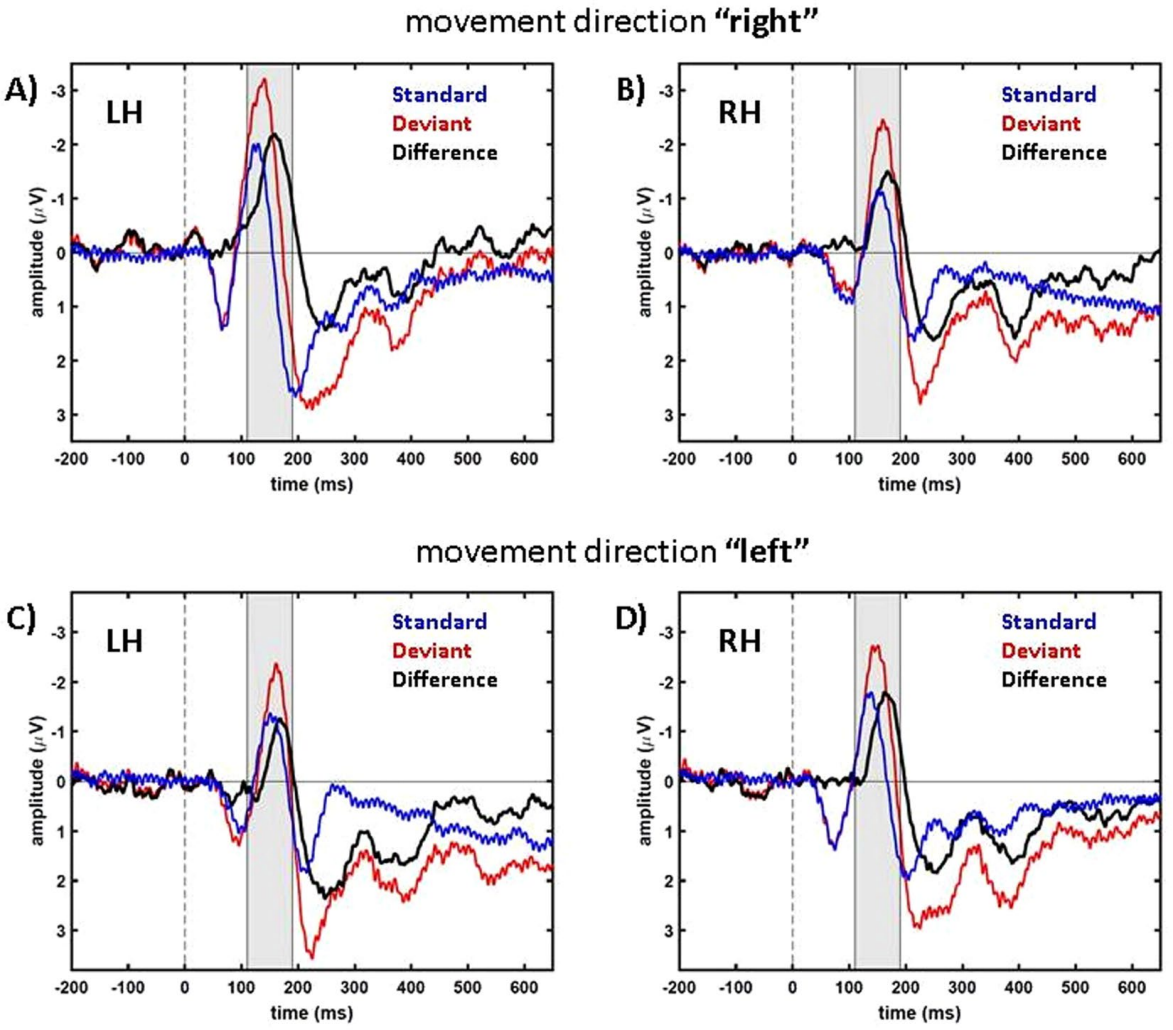
Average time courses of parieto-occipital electrodes in the “central” condition. In blue, data of standard trials are presented, in red, data of deviant trials, and in black the differences (standard data subtracted from deviant data). (**A**,**B**) show the condition “right” (i.e. target reappearance in the right visual hemifield) and (**C**,**D**) the condition “left” (reappearance in the left visual hemifield). In (**A**,**C**) data show averages across the electrode cluster LH (P3, P7 and PO3). Accordingly, in (**B**,**D**) data show averages from electrode cluster RH (P4, P8 and PO4). Thus, panels (A,D) show signals contralateral to the side of the target’s reappearance while panels (B,C) show signals for ipsilateral reappearance. The gray areas mark the time window shown in Fig. 1.

Figure 2 shows the recorded EEG-signals not in topographic maps, but as time courses of voltages recorded from a single electrode cluster over time. In line with previous work^6,10^, we depict negative values upward in our data figures. In Fig. 2 the time courses of the deviant and standard data, as well as the difference between them (standard subtracted from deviant) are plotted. In all cases, we pooled data from three electrodes positioned either over the left hemisphere (electrodes P3, P7 and PO3; called electrode cluster LH) or the right hemisphere (electrodes P4, P8 and PO4; cluster RH). The more negative N2-component in the ERP for deviant (red) as compared to standard stimuli (blue) results in a negative peak between 110 ms and 200 ms in the difference signal (black) corresponding to the MMN.

For our main statistical tests, we analyzed average ERP values of the respective contralateral LH or RH electrode cluster in the time window between 110 ms and 190 ms (indicated by the gray shaded area in Fig. 2). As shown in Figs 1 and 2, this temporal window centers on the time of the peak of the MMN.

We calculated t-tests for this time window to test our hypothesis that an MMN-component can be observed on electrodes contralateral to the stimulated visual hemifield where a more direct response to the visual stimulation can be expected^32^. We calculated one-sample t-tests to determine if the components were significantly different from 0 µV and also paired t-tests to compare the different conditions. Accordingly, for these 6 paired t-tests, p values had to be <0.0083 to indicate statistically significant differences (Bonferroni correction).

The time courses of the difference signal presented in Fig. 2 show negativities over both hemispheres, but the ERP-components were significantly stronger (more negative), i.e. more pronounced, for the contralateral as compared to the ipsilateral hemisphere, both relative to the side of the target reappearance (comparison between RH and LH signals for the different motion directions: “right”: n = 8. Paired t-test: *t*(7) = −4.17, *p* = 0.0042 and “left”: *t*(7) = −4.33, *p* = 0.0035). In the following we will analyze the pronounced contralateral ERP-components only because they show the more direct response to the visual stimulation compared to the ipsilateral brain hemisphere which obtains for the most part indirect visual input via the corpus callosum^32^.

For both motion directions, “right” and “left”, the difference between standard and deviant trials resulted in a negativity-component that is visualized in Figs 1 and 2 and which was statistically significant different from 0 µV for our group of subjects (n = 8. One-sample, one-tailed t-test: condition “right”, electrode cluster LH: *t*(7) = −7.94, *p* = 0.00005; condition “left”, electrode cluster RH: *t*(7) = −3.85, *p* = 0.0032).

**Figure 3:**
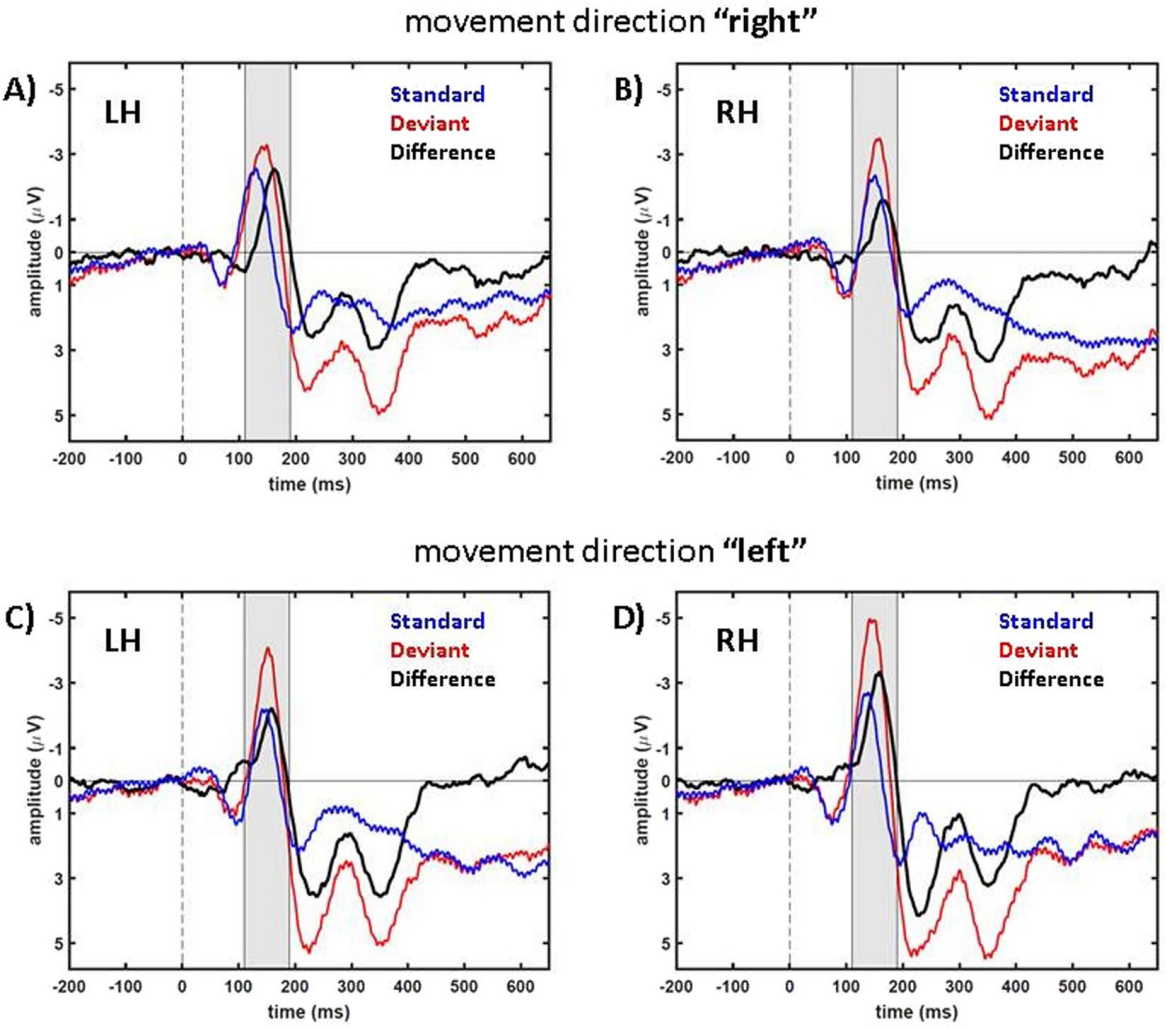
Population data from condition “peripheral”. In blue data of standard trials are presented, in red data of deviant trials and in black the differences (standard data subtracted from deviant data) are shown. (**A**,**B)** show condition “right” and (**C**,**D**) condition “left”. In (**A**,**C**) data from LH is presented and in (**B**,**D**) data from RH.

We found comparable results for both attentional conditions. Figure 3 shows the respective data of the “peripheral” condition. Also here, the MMN effect was significant over contralateral parieto-occipital electrodes (n = 8. One-sample, one-tailed t-test: condition “right”, electrode cluster LH: *t*(7) = −4.99, *p* = 0.0008 and condition “left”, electrode cluster RH: *t*(7) = −4.13, *p* = 0.0022). MMN amplitudes over LH and RH were significantly different for motion direction left (comparison RH and LH: n = 8. Paired t-test: “right”: *t*(7) = −1.99, *p* = 0.087; “left”: *t*(7) = −4.36, *p* = 0.0033). The MMN-components of the two attention conditions were not significantly different for motion condition”right” but for motion condition “left” (motion condition “right”, electrode cluster LH: *t*(7) = −1.2, *p* = 0.27; motion condition “left”, electrode cluster RH: *t*(7) = 2.9, *p* = 0.022. Paired t-test for the time window 110 ms–190 ms, n = 8).

In addition to the MMN, we also observed a positivity in the difference data (black) from about 200 ms to 400 ms with two apparent peaks around 225 ms and 350 ms. This effect peaked on electrode Cz. An analysis of the average ERP at this electrode revealed that the positive component was significantly different from 0 µV for both motion directions (attention condition “central”, motion direction “right”: *t*(7) = 3.17, *p* = 0.0079; motion direction “left”: *t*(7) = 5.05, *p* = 0.0007; attention condition “peripheral”, motion direction “right”: *t*(7) = 6.47, *p* = 0.0002; motion direction “left”: *t*(7) = 8.36, *p* = 0.00003; one-sample, one-tailed t-test for the time window 200 ms – 400 ms). This component showed a significant difference between the two attention conditions (motion direction “right”: *t*(7) = −3.22, *p* = 0.015; motion direction “left”: *t*(7) = −2.85, *p* = 0.025; paired t-test for the time window 200 ms–400 ms).

### The effects of target predictability

In addition to investigating the effects of prediction error, we also aimed to characterize the impact of predictability of visual motion processing. To this end, we compared ERPs elicited by the reappearance of the moving target in the standard trials of the “full trajectory” condition to appearance of the moving target, i.e. data measured in the condition “half trajectory, appear”. In both cases, we aligned the data to the (re)appearance of the target. Importantly, visual stimuli were physically identical.

**Figure 4:**
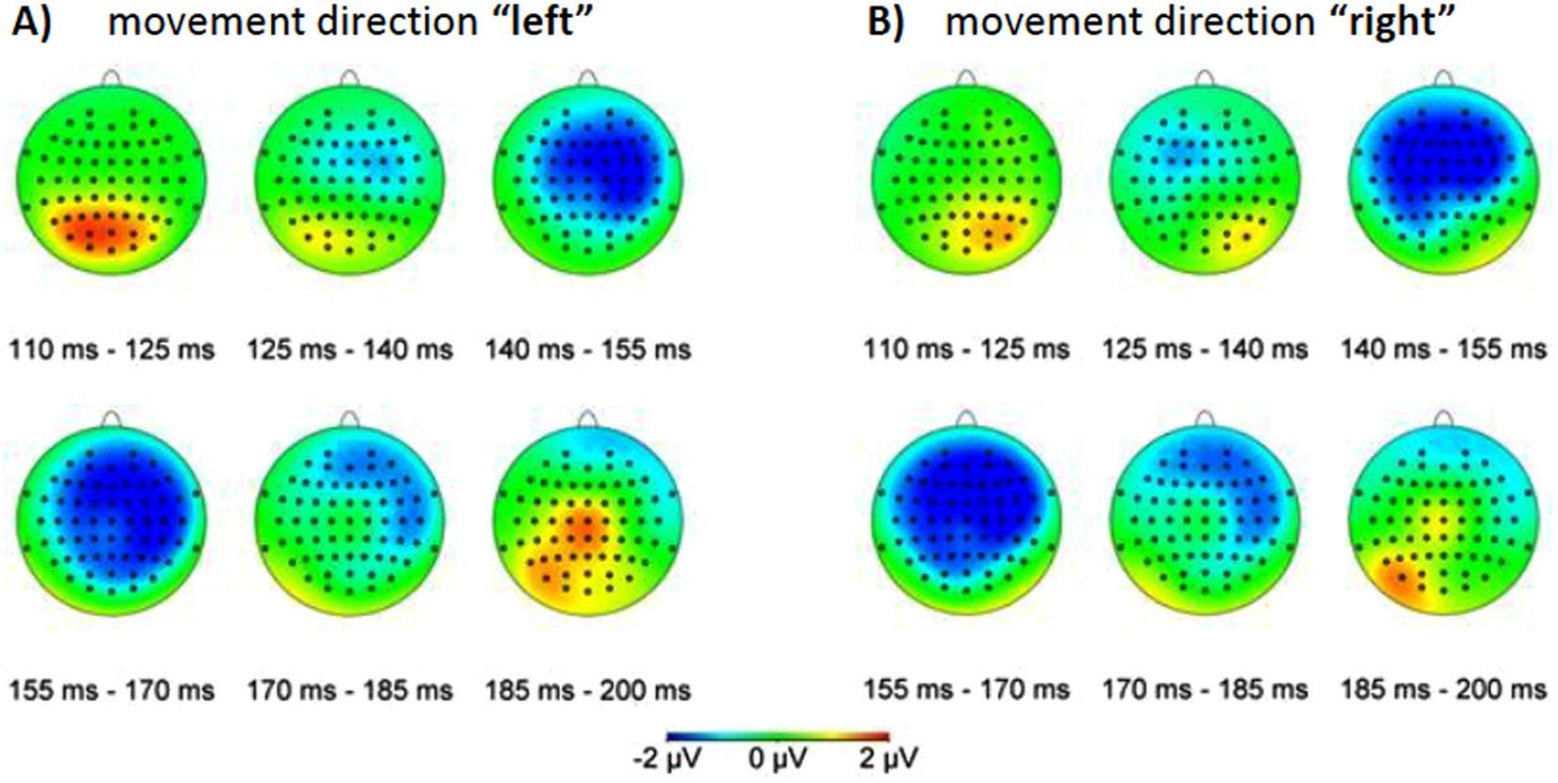
Difference between “complete trajectory, standard trials” and “half trajectory, appear” data. Each plot shows the ERPs averaged over 15 ms from trials in which subjects attended to the central target. Panel (A) shows data for stimuli presented in the left half of the visual field and panel (B) for stimuli presented in the right half of the visual field.

Figure 4 shows as topographic maps the ERP differences (“complete trajectory” of standard trials triggered on reappearance subtracted from “half trajectory, appear” data) between these two conditions while subjects attended the central fixation target (“central”). We found three main response components: (i) a positivity around 110 ms to 130 ms over parieto-occipital electrodes ipsilateral to the stimulus presentation. (ii) a negative component of the ERP difference signal between 140 ms and 170 ms over frontal (contralateral) and central electrodes. (iii) a positivity over left temporo-occipital and central electrodes. For both motion directions, this latter temporo-occipital component of the ERP signal was found over the left hemisphere only.

**Figure 5:**
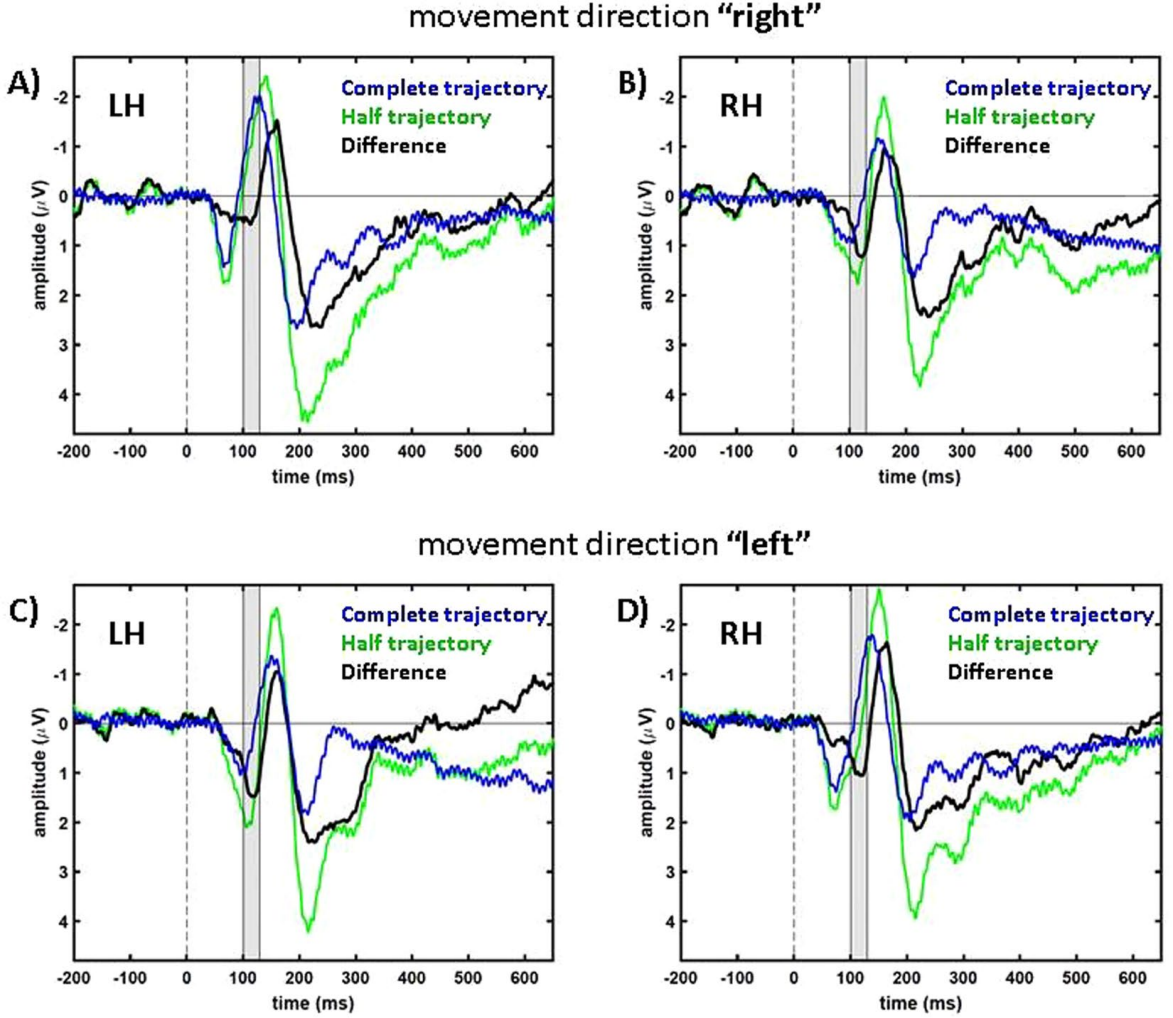
Signals with respect to the motion directions of posterior electrodes in condition “central”. In blue data of the second motion phase of “complete trajectory” trials are presented, in green data of “half trajectory, appear” trials and in black the differences (“complete trajectory”, 2. Phase of standard trials subtracted from “half trajectory, appear” data) are shown. (**A**,**B**) show condition “right” and (**C**,**D**) condition “left”. In (**A**,**C**) data from LH is presented, in (**B**,**D**) data from RH. The gray area marks the times that were analyzed for this electrode cluster, i.e. 100 ms to 130 ms.

**Figure 6:**
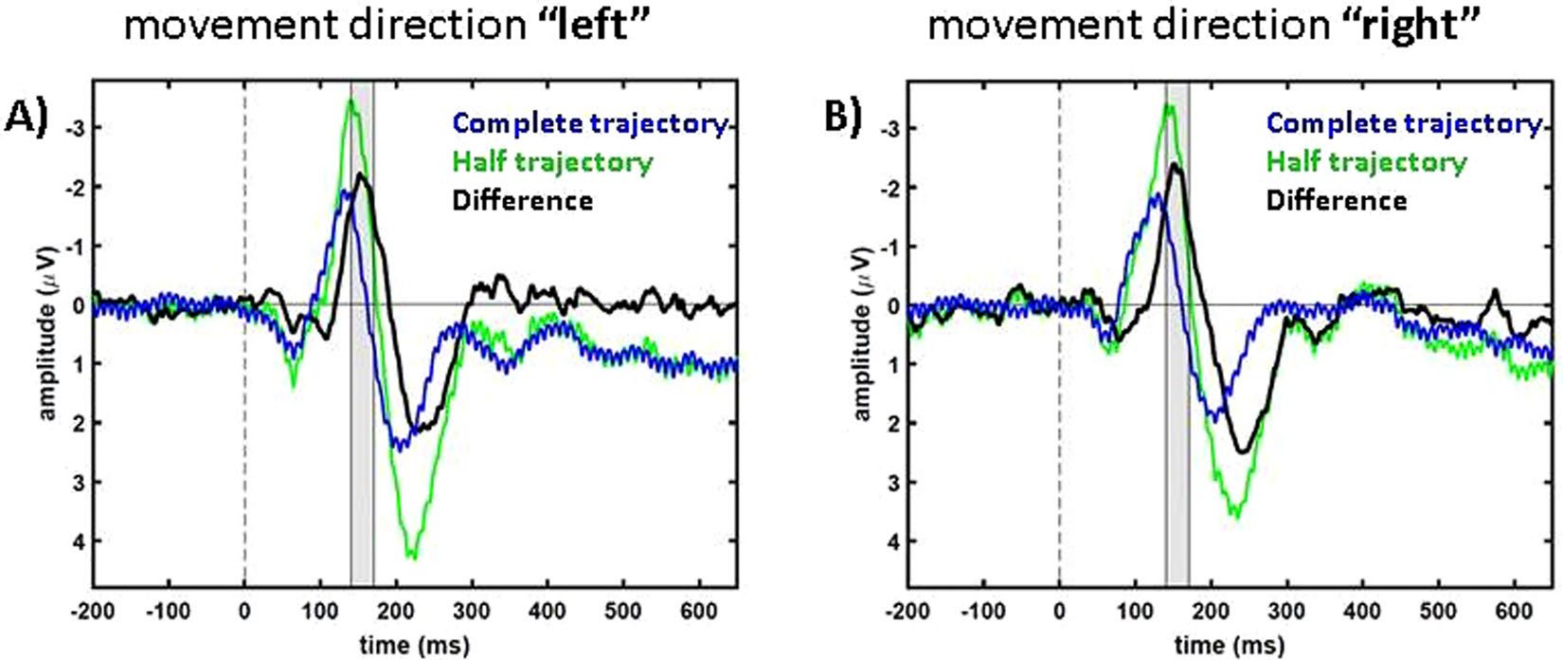
Signals with respect to the motion directions of frontal electrodes in condition “central”. In blue data of the second motion phase of “complete trajectory” trials are presented, in green data of “half trajectory, appear” trials and in black the differences (“complete trajectory”, 2. Phase of standard trials subtracted from “half trajectory, appear” data) are shown. (**A**) shows condition “left” and (**B**) condition “right”. In (**A**) data pooled over electrodes over the right hemisphere (F4, FC2, FC6) is presented, in (**B**) data pooled over electrodes over the left hemisphere (F3, FC1, FC5). The gray area marks the times that were analyzed for this electrode cluster, i.e. 140 ms to 170 ms.

**Figure 7:**
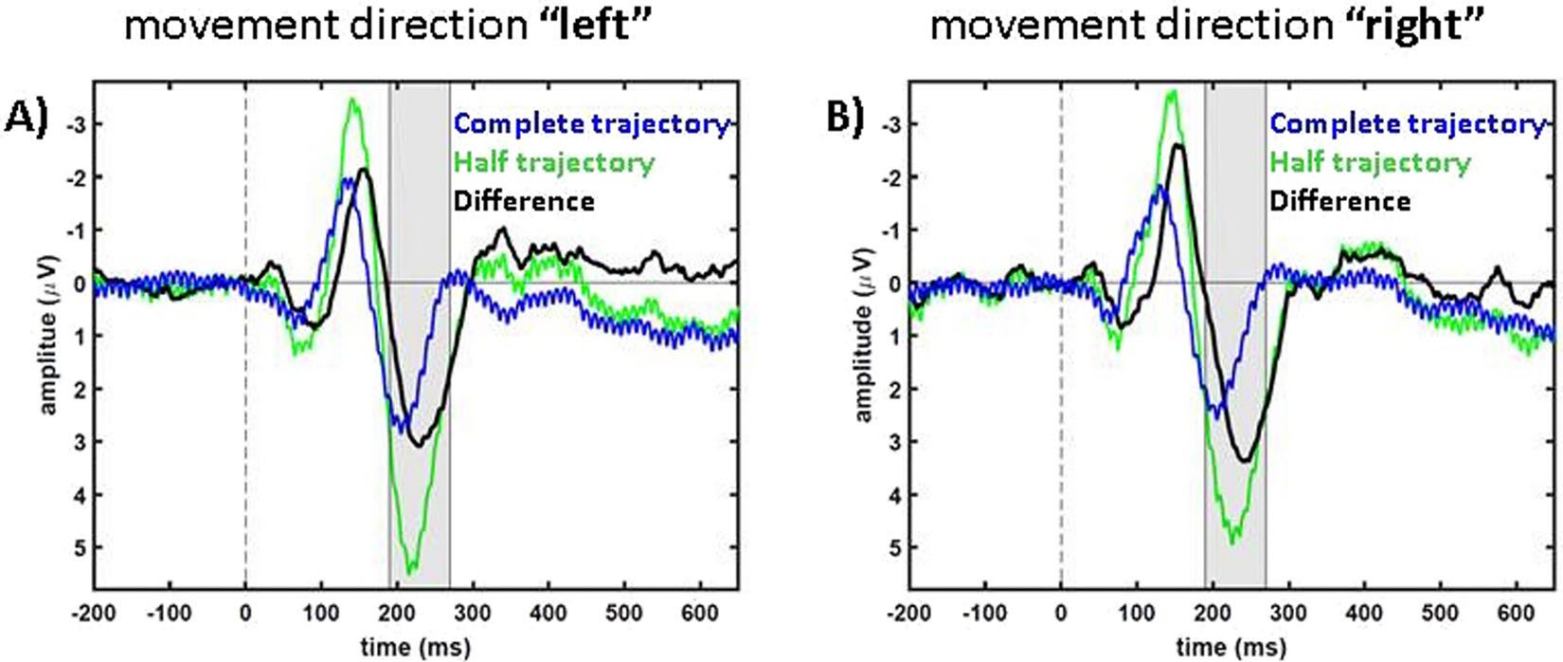
Signals with respect to the motion directions of central electrodes in condition “central”. In blue data of the second motion phase of “complete trajectory” trials are presented, in green data of “half trajectory, appear” trials and in black the differences (“complete trajectory”, 2. Phase of standard trials subtracted from “half trajectory, appear” data) are shown. (**A**) shows condition “left” and (**B**) condition “right”. Data pooled over electrodes (Fz, FC1, FC2, Cz, FCz) is presented. The gray area marks the times that were analyzed for this electrode cluster, i.e. 190 ms to 270 ms.

For a more detailed analysis we extracted the signals as well as their difference for electrodes at the locations of the prior described components. The results are shown in Figs 5–7. Figure 5 shows results from the posterior electrode clusters LH (average over P3, P7 and PO3) and RH (average over P4, P8 and PO4). We applied a one-sample, one-tailed t-test on the data in a time window from 100 ms to 130 ms after stimulus presentation (gray shaded area in Fig. 5) to investigate the positive posterior component (RH for condition “right”: *t*(7) = 3.0, *p* = 0.01; and LH for condition “left”: *t*(7) = 4.22, *p* = 0.002) which is significantly different from 0 µV. The frontal negativity in the time window between 140 ms and 170 ms (gray shaded area in Fig. 6) was also significantly different from 0 µV for both motion directions over frontal electrodes on the contralateral sides (“right”, averaged over F3, FC1 and FC5: *t*(7) = −4.88, *p* = 0.0009; “left”, averaged over F4, FC2 and FC6: *t*(7) = −4.99, *p* = 0.0008). As a last step, we tested the late positivity averaged over central electrodes Fz, FC1, FC2, Cz and FCz in the time window from 190 ms to 270 ms: one-sample, one-tailed t-test: “right”: *t*(7) = 5.43, *p* = 0.0005; “left”: *t*(7) = 4.2, *p* = 0.002 (gray shaded area in Fig. 7). The three different components did not show a significant difference if we compared the results from the attention condition “central” with the attention condition “peripheral” with an exception of the first positive component for the motion direction “right” (first component: “right”, RH: *t*(7) = −2.44, *p* = 0.045; “left”, LH: *t*(7) = −1.11, *p* = 0.3; second component: “right”, frontal LH: *t*(7) = −0.81, *p* = 0.45; “left”, frontal RH: *t*(7) = 0.58, *p* = 0.58; third component: “right”, central: *t*(7) = 1.99, *p* = 0.09; “left”, central: *t*(7) = 1.66, *p* = 0.14).

## Discussion

In this study, we investigated preattentive mechanisms of visual motion processing as well as the role of predictability on the processing of target trajectories using human EEG. First, we examined if motion trajectories are processed preattentively by probing for a visual mismatch negativity (MMN). In all trials, a visual stimulus moved along a horizontal trajectory. In ‘‘complete trajectory’’ trials, stimuli moved across the whole screen but were temporarily occluded. After reappearance, stimuli moved either along the same linear trajectory (*standard trials*) or at a different trajectory (*deviant trials*). Although the participants’ attention was always captured by a behavioral task, and the trajectory of the moving target was irrelevant to the task, we found a strong visual MMN for deviant trials. As the MMN was largely unaffected by the subjects’ focus of attention, processing of trajectories can, therefore, be considered to be preattentive^20,24–26^. We are going to discuss this finding for data collected in both our behavioral tasks together because the task did not induce a significant difference.

Second, we asked if the processing of non-predictable visual motion onsets is different for predictable vs. non-predictable motion onset. In a subset of trials, targets moved only half a trajectory, either as disappearing or re-appearing stimuli (“half trajectory” trials). We compared EEG-responses for re-appearing “half trajectory” trials with the EEG-responses of the second phase of complete trajectory trials (only from standard trials). We found three significant response differences, separated in time and space.

It is important to note that we compared in this study only ERPs elicited by physically identical stimuli. The design of our experiment allowed us to present exactly the same stimuli in different contexts, i.e. either as standard or as deviant stimulus as well as predictable or non-predictable stimulus. Our results can therefore not be explained by any physical differences of the visual stimuli.

Due to the fact that the target was not visible before it started its motion, the stimulus we presented in this study was a mixture of a flash-like and a motion-onset stimulus. Therefore, we expected the data to show the characteristics of both stimulus types described in earlier studies (for flash stimuli^33^ and for motion-onset stimuli^34^). Our ERP data are in line with this hypothesis. Firstly, all typical flash-ERP-components were present in our data (see Figs 2 and 3) that have been described earlier^33^. And secondly, the components P1, N2 and P2 were clearly pronounced in our data. This was to be expected for motion-onset ERPs^34^.

The MMN is not only considered to be a neural correlate of a perceptual prediction error^35^, but also to be indicative of preattentive processing of sensory stimuli^25^. In our study the difference between standard and deviant trials, i.e. the change in the target’s motion trajectory, induced such a prediction error. Accordingly, we hypothesized a larger N2-component in deviant trials as compared to standards^9,36^. Indeed, we observed a negative peak of the difference signal ERP_Deviant_ − ERP_Standard_ at around 150 ms after stimulus motion onset which is well in line with the expected time range for the peak of the MMN^3^. This negative component revealed an occipital-posterior scalp distribution as has been documented by other MMN studies^3,36^. In addition, the MMN-component could be observed most prominently over the hemisphere contralateral with respect to the stimulus appearance location. A larger N2-component over contralateral hemispheres has been reported before^28^.

In order to further test the preattentive characteristic of the MMN which was reported in previous studies^24^, the attention of the participants was controlled in two different behavioral tasks, i.e. with attention directed towards the fixation target or the moving stimuli. In both cases, the task (change in spatial frequency of the target) was not related to the signal which induced the MMN, i.e. the change in trajectory. As discussed in the results section, the behavioral performance of the participants clearly indicated their ability to attend to the instructed target. We found a significant difference of the MMNs in the two attention conditions for one motion direction (left) but not for the other (right). We had no a priori hypothesis concerning such an asymmetry of results. Importantly, we found a MMN for both attention conditions, central and peripheral, and both motion directions, left and right. Unexpectedly, the MMNs in the peripheral condition were significantly different for one motion direction (left). Accordingly, this difference within the peripheral attention condition might underlie the difference of the MMNs in the two attention conditions, central vs. peripheral, as also found for motion condition “left”. This consideration might suggest that the MMNs tended to be similar in both attention conditions. It is important to note, however, that the finding of a lack of MMN-differences in the two attention conditions does not prove that there are no such differences. This follows the general rule that the *absence of evidence* must not be mistaken as *evidence of absence*^37^. Instead, further experiments are required to resolve this issue.

Previous studies have shown that directions of linear motion can be misperceived^38–42^. At first glance, such a misperception might have had an influence on our results, in which we report a mismatch negativity in an oddball paradigm involving changes in movement trajectories. Yet, there are important differences between the studies by Tripathy and colleagues and our current work. In the Tripathy *et al*. studies^38–41^, the task of the subjects was to judge the direction of visual motion. In our study, however, the direction of motion was not relevant to the behavioral task. Instead, subjects had to attend to the occurrence of a change in spatial frequency of either a central or peripheral target. It is exactly this finding which reveals the preattentive nature of the processing of linear visual motion as indicated by the MMN. Furthermore, Tripathy and colleagues could show that misperceptions of direction typically occur only, when a number of elements move simultaneously, while in our study only one element moved at a time. Finally, it was a key aspect of our study that we compared EEG responses for physically identical stimuli. So, regardless of how a direction of visual motion would have appeared to the observers, it was the same in the standard and the oddball condition, whose comparison revealed the MMN.

Previous studies could show that large deviations in movement trajectories can be even missed entirely^39,41,42^. While exact experimental conditions were quite different in the studies by Tripathy and colleagues as compared to ours, these previous studies allow for a rough estimate of the threshold of trajectory change that might get missed. Based on Tripathy *et al*.^39^, this results in a change of clearly less than a 1° degree of visual angle that could have been missed by the observers. In our oddball condition, the trajectory changed from 4.2° below the horizontal meridian (HM) to 4.2° above the HM (or vice versa), i.e. the step-like change was larger than 8°. In other words, it is highly unlikely that trajectory changes were missed. In addition, our finding of a MMN can be considered a neural correlate of subjects not missing the trajectory change.

In the comparison between the deviant and standard trials we also consistently found a P300-component starting at about 200 ms after motion onset (Figs 2 and 3). Previous studies concluded that neuronal representations have to be updated following a deviant stimulus which results in a P300-component^43^. The P300-component is a robust effect that can be observed for different stimuli (auditory, visual or somatosensory) and different response and observation modalities of the experiments, like counting or button presses^44^. Considering these previous results, we expected to find a P300-component also in our experiment.

In our oddball paradigm, the trajectory of the second motion phase was implicitly predictable based on the trajectory prior to the occlusion, i.e. the first motion phase. The prediction was confirmed in standard trials and violated in deviant trials. In order to study the processing of visual motion at different predictability levels, we presented “half trajectory, appear” trials that showed only the second phase of a motion. While being physically identical to the second half of “standard trials”, “half trajectory, appear” trials did not allow participants to generate a prediction about the upcoming motion. Based on previous studies^29^, we expected a larger P1-component in the predicted “complete trajectory” trials as well as an attenuated N1-component and an increased P3-component in response to the unpredicted “half trajectory, appear” trials^45^.

Different to Doherty and colleagues^29^, we found a larger P1-component in trials in which the participants had the least information about the target trajectory, i.e. in trials of the condition “half trajectory, appear”. This difference might be due to different attentional states of the observers. Previous studies have shown that the P1-component is strongly influenced by attention^46,47^ but also arousal^48^. Accordingly, the larger P1-component as found in our study could be due to a higher level of arousal in the experimental condition with less information about the target trajectory.

In our study all ERP-components were larger for data from unpredicted trials. Especially we found a larger amplitude for the P3-components in the unpredicted “half trajectory, appear” trials compared with the predictable trials (Figs 4, 7) which is in line with previous studies^45^. This enhanced P3-component reveals an effect known to follow the earlier effects of prediction violation to update an internal model and is considered indicative of an enhanced processing of the unpredicted and, therefore, more surprising “half trajectory, appear” stimuli^45^.

Similar to the MMN which became apparent by the comparison of standard and deviant trial data, the comparison between trials of the condition “half trajectory, appear” and standard trials of the condition “complete trajectory” revealed a strong lateralized negativity in a time window from 140 ms to 170 ms. Interestingly, however, in contrast to the parieto-occipital focus of the MMN this component was centered over frontal areas (Fig. 4) suggesting more cognitive aspects to be involved in the processing of visual motion information^49^.

Given that the stimuli were physically identical in the half and full trajectory conditions, the observed differences in the ERPs must be due to internal processing. Most likely, participants generated an internal model of where the target should show up after passing the occluder, which was not possible in the half trajectory condition. The observed differences in the ERPs are indicative of this internal model, i.e. prediction.

Taken together, our results provide strong evidence for an influence of prediction and predictability on the processing of visual motion.

## Materials and Methods

### Participants

Eight subjects (six female, aged between 24 and 35, mean 27.6 years) participated in this study. They were right handed and had normal or corrected to normal vision. All participants, except for two (the authors CS and SK) were naïve about the purpose of the experiment and were compensated with 6 € per hour for participation. The participants performed the experiment on four different days, except for one participant, who came on five different days. Due to technical issues, we could not complete the data collection with this participant on one of the days and had to invite this participant for a fifth day. The study was approved by the Ethics Committee of the Faculty of Psychology at Philipps-University Marburg and was in agreement with the Declaration of Helsinki. Before the experiment all participants provided written informed consent. After completing the experiment interested participants were given full disclosure on the purpose of the experiment.

### Setup

Participants sat on a chair in a darkened, sound attenuated and electrically shielded room. They rested their head on a chin rest, placed centrally in front of a screen. The distance between the screen and the participant’s eyes was 71 cm. The screen (SyncMaster, Samsung, Seoul, South Korea) was a 40 cm (31°) wide and 30 cm (24°) high CRT display. The resolution of the screen was set to 1152 × 864 pixels and the refresh rate was 100 Hz.

### Stimulus

**Figure 8:**
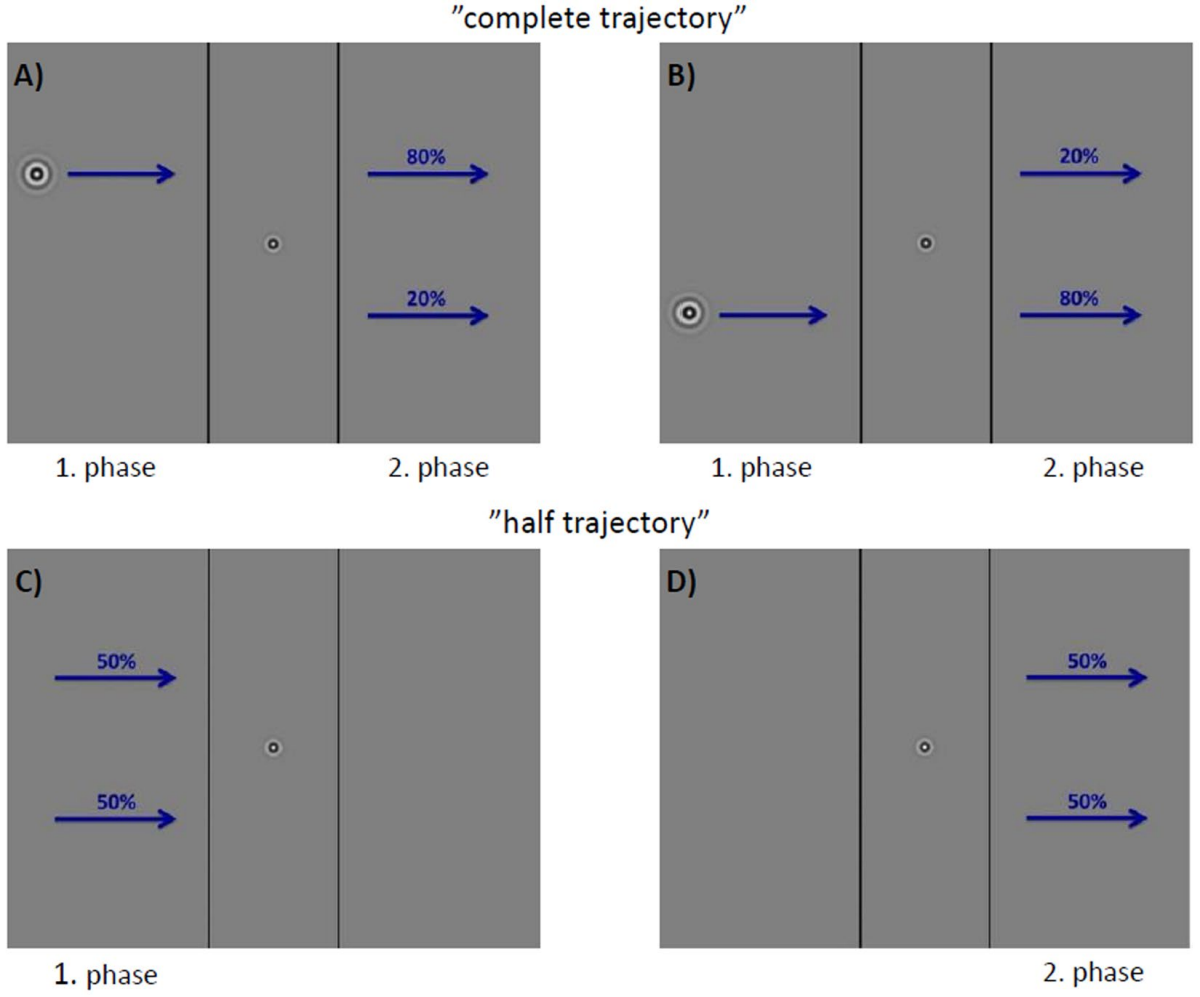
Examples of the possible target trajectories for the motion condition “right”. Between the two black lines the target was occluded and invisible to the participants. (**A**,**B**) show the condition “complete trajectory” that occurred in 5 out of 7 trials. Arrows marked with 80% indicate the standard and those marked with 20% the deviant stimuli. Condition “above” is shown in (**A**) while condition “below” is presented in (**B**). In (**C**,**D**) the condition “half trajectory” is presented. In (**C**) the condition “disappear” and in (**D**) the condition “appear” are schematically shown. In both cases an equal amount of trials was presented at the upper and at the lower trajectory (50%). Condition “disappear” and “appear” were each presented with a ratio of 1 out of 7. In order to control the focus of attention, the participants performed a change detection task requiring them to report changes in spatial frequency of either the central fixation target (“center”) or the peripheral motion target (“peripheral”; c.f. Methods).

The stimulus, illustrated in Fig. 8, consisted of a horizontally moving target (detailed description see below) on a gray screen (luminance: 29 cd/m^2^). In the central part of the screen the motion of the target was covered by an occluder that was marked by two vertical black lines. The width of the occluder changed after each block, i.e. a set of 20 trials, and varied pseudorandomly between 7.1° and 10.6°. It was chosen such that the time between disappearance and reappearance was long enough such that the ERPs evoked by the disappearing target did not interfere with the ERPs upon reappearance; given its steady velocity of 15.2°/s, the target was occluded for intervals between 400 ms to 600 ms duration. Upon (re)appearance, the whole target was first completely visible after about 210 ms.

The moving target was a concentric black and white Gabor pattern (sine wave in polar coordinates × Gaussian distribution with a standard deviation of sigma = 15 pixels). The tail of the Gabor pattern was cut such that the diameter of the pattern was 3.2°. In the center of the screen a stationary fixation target was visible throughout each trial. This fixation target was a smaller version of the moving concentric Gabor pattern with a diameter of 1.6°.

We used four different conditions to describe the motion trajectory of the target. For each trial, the combination of a direction and a position condition were chosen pseudorandomly: motion to the left (“left”), motion to the right (“right”), target trajectory 4.2° above the fixation point (“above”) and target trajectory 4.2° below the fixation point (“below”).

In the condition “complete trajectory” (5 out of 7 trials) the target appeared on one side of the screen and moved across the whole screen. Accordingly, this motion contained an occlusion phase in the middle of the screen which divided it into two motion phases, a first one before and a second one after the occlusion. These two phases were important for the Oddball-experiment because the second phase defined the difference between standard and deviant trials. In standard trials the trajectory did not change but was continuous whereas in deviant trials the target changed its trajectory from “above” to “below” or *vice versa (*dis-continuous). This switch happened in the middle of the motion while the target was occluded. In 80% of the trials a standard stimulus was presented and a deviant stimulus in 20% of the trials. The first phase of these “complete trajectory” trials could be used to create an (implicit) prediction about the trajectory in the second phase. Importantly, due to the balanced stimulus design, the second motion phases from standard and deviant trials were physically identical and differed only because of their first motion phases.

In “half trajectory” trials (2 out of 7 trials) the target moved only across half of the monitor width. This resulted from two different conditions: The target could move from one edge of the screen to the occluder and disappear behind it (“disappear” condition, tested in 1 out of 7 trials). In the second condition, the target started in the middle of the screen and moved to one of the monitor’s edges (“appear”, in 1 out of 7 trials).

### Task

To control the focus of the participants’ attention we ran two different attention conditions. While fixating the center of the screen, the subjects had to attend either the central fixation target, “central”, or the moving target, “peripheral”. Participants were told before the start of the experiment which target (central vs. peripheral) was to be attended. In any case, participants had to fixate the central target throughout each trial. Simultaneously, they were asked to press the key “5” on the numerical pad of a keyboard in front of them when the spatial frequency in the attended target (central or peripheral) changed, which happened in 10% of all trials. They appeared randomly in a time window from 300 ms after target appearance to 300 ms before target disappearance. After a change the targets retained the new spatial frequency throughout the rest of the trial. Participants were instructed to press the key as fast as possible once they became aware of the change. After the completion of a key-press-trial participants further had to report whether the spatial frequency had been scaled up (press key “2”) or down (press key “8”). Participants answered using their right or left hand. The response hand (left vs. right) was pseudo-randomly assigned within one session. Trials that showed a change in spatial frequency were later excluded from data analysis. We collected the data for each participant on four different days, with one exception as detailed in the section *participants*. On each day only one attention condition was employed, and we always showed the condition “central” on the first day. We considered this the easiest condition for the participants and we wanted them to get used to the paradigm. The conditions were pseudorandomized for the other three days to minimize effects of the presentation order in our data.

### Procedure

In total, 448 blocks with 20 trials were run in each participant corresponding to a total of 4480 trials for each of the two attention conditions. One trial consisted of a motion phase (2 s for “complete trajectory”, 1 s for “half trajectory”) and an inter trial interval of a pseudorandomly chosen length (between 0 ms and 400 ms). The resulting total measuring time per subject was about 8 hours and the required data was typically collected in four sessions. In a given block, the conditions (“complete trajectory”, “half trajectory, appear” and “half trajectory, disappear”) as well as the width of the occluder were fixed. The motion direction (conditions “right” and “left”) was pseudorandomly chosen for each block.

### EEG recordings

The electroencephalogram (EEG) was continuously recorded with an actiCHamp module and Brain Vision PyCorder (*Brain Vision LLC*, Morisville, NC, USA). An electrode cap with 64-channel Ag/AgCl active electrodes was used. Electrodes were positioned according to the extended international 10–20 system. Additionally, five electrodes were fixed on the skin to measure the electrooculogram (EOG). The Vertical EOG was recorded with two electrodes above and below the left eye. The Horizontal EOG was recoded with two electrodes placed on the outer canthi of both eyes. The fifth EOG electrode served as ground electrode and was fixed to the forehead. The impedances of all electrodes were principally kept below 5 kΩ throughout the experiment. EEG and EOG signals were recorded as continuous signals digitized at a sampling rate of 1000 Hz.

### Analysis

The offline analysis was conducted with Brain Vision Analyzer software (*Brain Products*, Gilching, Germany). The average signal of the mastoid electrodes TP9 and TP10 was used as the reference signal. Data were low pass filtered with a cut-off frequency of 70 Hz and a Notch filter at 50 Hz was applied. Trials with blinks or eye movements (18% of all trials) were excluded from further analysis as well as trials in which a change in the spatial frequency of the Gabor pattern occurred and, therefore, a motor response of the participants was induced. All data were aligned to the (re)appearance of the moving target next to the central occluder, termed stimulus onset in the following. For each electrode, EEG epochs from 200 ms before until 700 ms after stimulus onset were extracted from the continuous data stream. Each epoch was baseline corrected (centered) using the average signal from −50 ms to 0 ms and epochs were averaged for subjects and conditions separately.

To calculate the MMN-component, only data collected in the condition “complete trajectory” were used. The MMN is defined as the difference of ERPs for deviant and standard stimuli^6^ (i.e. ERPs from standard stimulus trials have to be subtracted from ERPs elicited by deviant stimulus trials). For this subtraction, we only used data with completely identical physical properties in the second motion phase, but different context, i.e. a given stimulus served as deviant in one condition and as standard in the other condition. For our statistical analyses, we aimed to compare ERP values from a fixed temporal window across all conditions. To this end, we first determined the time point of the peak of the MMN and the maximum width to cover its full time course. This resulted in an analysis window ranging from 110 ms to 190 ms after stimulus onset.

For our statistical tests (paired or one-sample t-tests), we always employed a threshold criterion of p < 0.05 to determine statistical significance.

The datasets generated and analyzed during the current study are available from the corresponding author on reasonable request.
